# Luminescence- and nanoparticle-mediated increase of light absorption by photoreceptor cells: Converting UV light to visible light

**DOI:** 10.1038/srep20821

**Published:** 2016-02-10

**Authors:** Lei Li, Sunil K. Sahi, Mingying Peng, Eric B. Lee, Lun Ma, Jennifer L. Wojtowicz, John H. Malin, Wei Chen

**Affiliations:** 1 Department of Biological Sciences, University of Notre Dame, Notre Dame, IN 46556, USA; 2 Department of Physics, and Center for Security Advances via Applied Nanotechnology, University of Texas, Arlington, TX 76019, USA; 3 The State Key Laboratory of Luminescent Materials and Devices, South China University of Technology, Guangzhou, China 510641

## Abstract

We developed new optic devices – singly-doped luminescence glasses and nanoparticle-coated lenses that convert UV light to visible light – for improvement of visual system functions. Tb^3+^ or Eu^3+^ singly-doped borate glasses or CdS-quantum dot (CdS-QD) coated lenses efficiently convert UV light to 542 nm or 613 nm wavelength narrow-band green or red light, or wide-spectrum white light, and thereby provide extra visible light to the eye. In zebrafish (wild-type larvae and adult control animals, retinal degeneration mutants, and light-induced photoreceptor cell degeneration models), the use of Tb^3+^ or Eu^3+^ doped luminescence glass or CdS-QD coated glass lenses provide additional visible light to the rod and cone photoreceptor cells, and thereby improve the visual system functions. The data provide proof-of-concept for the future development of optic devices for improvement of visual system functions in patients who suffer from photoreceptor cell degeneration or related retinal diseases.

In humans, retinal photoreceptor cells do not regenerate after apoptosis nor do they self-repair in response to damage (e.g., due to aging, gene mutation, or eye injury)^1^,^2^,^3^. While other types of light-sensitive neurons (e.g., melanopsin-containing ganglion cells) are present in the retina, they do not participate in visual imaging; instead they play a role in regulation of the circadian rhythms of animal physiology and certain types of visual reflexes such as pupillary constriction^4^,^5^,^6^. In patients who suffer from photoreceptor cell degeneration, the visual sensitivity is decreased.

One of the strategies for recovering the lost visual system functions in degenerating retinas is to increase the absorption of light by the remaining photoreceptor cells. In this research, we tested a hypothesis that the increase of light absorption (i.e., by converting UV light to visible light) may improve the visual system functions in control and eye-injured animals. Using singly-doped luminescence glasses and nanoparticle-coated lenses, we converted UV light to mid- and long-wavelength green or red light or wide-spectrum white light. We tested the effect of converted light in visual performance in zebrafish (wild-type controls, retinal degeneration mutants, and light-induced photoreceptor cell apoptosis models) using different types of behavioral assays. The data provide direct evidence for the usefulness of converted light (from UV light) for visual improvement in vertebrate animals.

## Results

**Figure 1:**
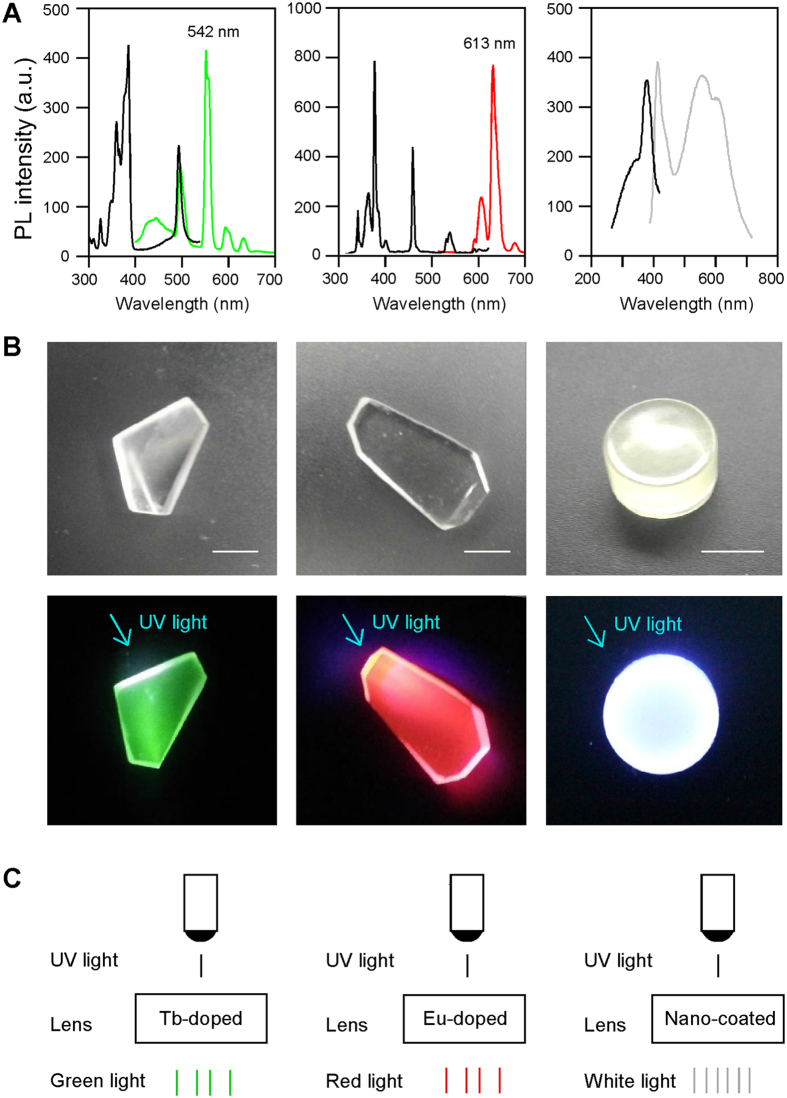
Conversion of UV light to mid- or long-wavelength green or red light or wide-spectrum white light by Tb^3+^ or Eu^3+^ singly-doped glasses or CdS-QD coated lenses. (**A)** Spectra of excitation light (UV light, black lines; peak spectrum 390 nm) and emission light (green, red and white light; color lines) by Tb^3+^ (left) or Eu^3+^ (middle) doped glasses, or CdS-QD coated lenses (right). Note that the UV source has different energy levels due to the crystal field of light bulbs and the excitation centers, which resulted in the appearance of additional small spectrum peaks. (**B**) Photographs of Tb^3+^ (left) and Eu^3+^ (middle) doped glasses, and CdS-QDs (right) coated lenses under normal room light (top panels) or after illumination with UV light in the dark (bottom panels). The intensity of UV light (maximum intensity, 50 lux) attenuated approximately 1 log unit (to 5 lux) after conversion. Scale bars: 1 cm. (**C**) Diagrams showing the conversion of UV light to green, red and white light through singly-doped luminescence glasses or nanoparticle-coated lenses.

We fabricated Tb^3+^ or Eu^3+^ singly-doped borate glasses and CdS-quantum dot (CdS-QD) nanoparticle-coated lenses that convert UV light to visible light which can be absorbed by human photoreceptor cells (human photoreceptor cells do not absorb UV light emitted from sunlight). Tb^3+^ or Eu^3+^ singly-doped borate glasses or CdS-QDs coated lenses are highly luminescent and transparent/translucent (Fig. 1A,B). They are cost-effective, easy to synthesize, and readily embedded or coated onto glasses^7^,^8^,^9^. When illuminated by a UV light source, they convert UV light to narrow-band green or red light, or wide-spectrum white light (Fig. 1C). The intensity of UV light attenuated approximately 1.0 log unit after conversion through the singly- doped glasses or nanoparticle-coated lenses.

**Figure 2:**
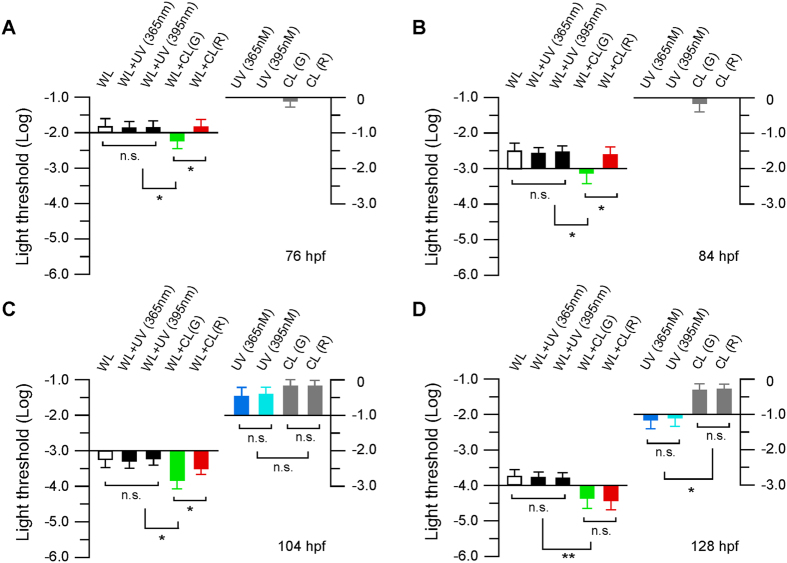
Behavioral visual threshold of zebrafish larvae tested under different lighting conditions. (**A,B**) Light threshold required to evoke the startle responses in 76 hpf and 84 hpf larvae. Under white light illumination, the startle responses were observed in all the animals examined. Note the decrease of light threshold after the addition of Tb^3+^ doped glass-converted green light at both time points. The addition of Eu^3+^ doped glass-converted red light produced no effect on the light threshold. Solo application of UV light or converted red light did not trigger the startle responses at either time point. However, solo application of converted green light triggered the startle responses when applied at or near the maximum intensity levels. (**C,D**) Light threshold required to evoke the OKR in 104 hpf and 128 hpf larvae. The OKR was observed in all the larvae examined. Note the decrease of light threshold after the addition of luminescence glass-converted green or red light. Sole application of UV light or converted green or red light also triggered the OKR. Data represent the Means  ±  SE (n = 12), *p < 0.05; **p < 0.01; n.s., not significant. Abbreviations: WL, white light; UV, ultraviolet light; CL(G), converted green light; CL(R), converted red light.

We examined the effect of converted light (after passing through singly-doped luminescence glasses or nanoparticle-coated lenses) on visual behaviors in control and eye-injured zebrafish models. Zebrafish retinas contain both the rod and cone photoreceptor cells^10^,^11^. In developing zebrafish, the retina is formed at 20 hours post fertilization (hpf) and the first rhodopsin protein expression can be detected at approximately 50 hpf^12^,^13^. Light-induced behaviors, such as the startle responses, can be recorded as early as 68 hpf, i.e., in response to abrupt light illumination, the embryo twists its body and initiate a movement^14^. We measured the light threshold required to evoke the startle responses in dark-adapted zebrafish larvae at 76 hpf and 84 hpf, respectively, during which stages the zebrafish visual sensitivity is largely mediated by the rod photoreceptor cells^15^,^16^,^17^. When tested with white light (Tungsten light; maximum intensity 1200 lux; the light intensity that illuminate the fish was adjusted using neutral density filters), the light threshold required to evoke the startle responses was −1.82 ± 0.21 and −2.51 ± 0.21 log units at 76 and 84 hpf, respectively (Fig. 2A,B). While the addition of UV light (Deuterium light, 365 nm or 395 nm wavelength; maximum intensity, 50 lux) produced no effect on the light threshold required to evoke the startle responses, when UV light was converted to narrow-band green light by Tb^3+^ doped glasses and co-applied with white light, increases of light sensitivity were observed, i.e., the light threshold that evoked the startle responses decreased to −2.23 ± 0.20 and −3.12 ± 0.29 log units (Fig. 2A,B). The addition of Eu^3+^ doped glass-converted red light produced no effect on the light threshold at either time point because the cone system circuits have not been formed during these stages of development. However, solo application of Tb^3+^ doped glass-converted green light triggered the startle responses (Fig. 2A,B). This is likely due to activation of the rod photoreceptor cells and related circuits, i.e., rod photoreceptor cells are sensitive to green light but not red light^18^.

By 4 days old, complex visual behaviors, such as the optic kinetic responses (OKR; in which the zebrafish larvae rotate their eyes following the directions of vertical strips that move across the receptive fields) can be recorded^19^. To further evaluate the effect of converted light in mediating animal visual behaviors, we measured the OKR in dark-adapted zebrafish larvae at 104 hpf and 128 hpf, respectively, during which developmental stages the cone system functions become evident. At 104 hpf, the light threshold required to evoke the OKR was −3.24 ± 0.20 log units (Fig. 2C). The addition of UV light produced no changes in absolute light threshold for the OKR. However, when UV light was converted to narrow-band green light and co-applied with white light, significant increases in light sensitivity were observed, i.e., the light threshold required to evoke the OKR decreased to −3.82 ± 0.22 log units (Fig. 2C). No significant changes in light threshold were observed by co-applying Eu^3+^ doped glass-converted red light and white light. Solo application of UV light or luminescence glass-converted green or red light also triggered the OKR (Fig. 2C), likely by activation of the UV cones and other cone cell types^20^. At 128 hpf, the larvae became more sensitive to light, and the light threshold required to evoke the OKR decreased to −3.72 ± 0.20 log units when illuminated by white light (Fig. 2D). The addition of UV light produced no significant changes in the light threshold for the OKR, but when the larvae were illuminated with white light and luminescence glass-converted green or red light, significant increases in visual sensitivity were observed, i.e., the light threshold required to evoke the OKR decreased to −4.36 ± 0.29 and −4.42 ± 0.80 log units, respectively (Fig. 2D). The OKR was also observed when the larvae were illuminated with UV light, either unfiltered or after conversion by Tb^3+^ or Eu^3+^ singly-doped glasses (Fig. 2D). This is due to the activation of the UV cones (by unfiltered UV light) or other cone cell types (by converted light).

To investigate if the converted light may have a role in improving the visual system functions in animals with eye injuries, we measured the light threshold required to trigger visually-mediated escape responses^21^ in adult zebrafish. The experiments were conducted in both the control and experimental groups, i.e., in wild-type fish maintained in normal lighting conditions and in zebrafish that underwent degeneration of rod and/or cone photoreceptor cells (e.g., *night blindness d* mutants, light-induced photoreceptor cell degeneration models)^22^,^23^.

**Figure 3:**
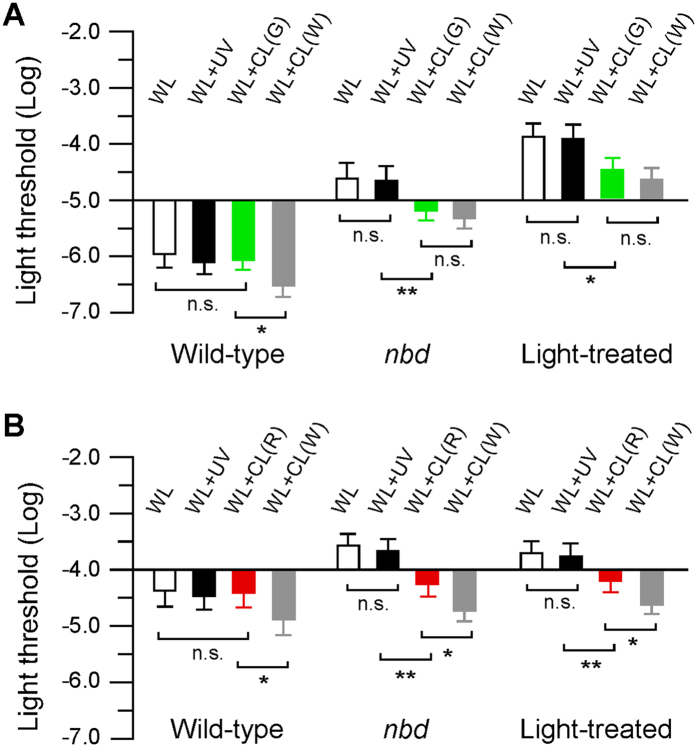
Behavioral rod and cone threshold of adult zebrafish (control, mutants, and eye-injured models) under different lighting conditions. (**A**) In dark-adapted control animals, the addition of UV light or Tb^3+^ doped glass-converted green light produced no effect on absolute rod threshold. The addition of CdS-QDs converted white light decreased the light threshold by half a log unit. In *nbd* mutants or light-treated retinal degeneration models, the addition of UV light produced no effect on the rod threshold. However, co-illumination of the fish with white light and Tb^3+^ doped glass-converted green light or CdS-QDs converted white light decreased the light threshold by nearly 1 log unit. (**B)** In light-adapted control animals, the addition of UV light or Eu^3+^ doped glass-converted red light produced no effect on the cone threshold, but the addition of CdS-QDs converted white light decreased the cone threshold by half a log unit. In *nbd* mutants or light-treated photoreceptor cell degeneration animals, the addition of luminescence glass-converted red light or nanoparticle-converted white light decreased the cone threshold by 1.0–1.5 log units. Data represent the Means ± SE (n = 12), *p < 0.05; **p < 0.01; n.s., not significant. Abbreviations: WL, white light; UV, ultraviolet light; CL(G), converted green light; CL(R), converted red light; CL(W), converted white light.

Visual thresholds (the minimum light required to evoke escape responses) were measured in both dark- (rod dominant) and light- (cone dominant) adapted animals. In control animals, under white light conditions, the absolute rod and cone threshold was −5.96 ± 0.22 and −4.39 ± 0.30 log units, respectively (Fig. 3A,B). While the addition of UV light or Tb^3+^ or Eu^3+^ doped glass-converted narrow-band green or red light produced no obvious effect on rod or cone sensitivity, co-illumination of the fish with white light and CdS-QDs converted white light led to the improvement of both the rod and cone system functions, i.e., the light threshold required to evoke escape responses decreased to −6.5 ± 0.19 log units (in dark-adapted animals) and −4.89 ± 0.26 log units (in light-adapted animals) (Fig. 3A,B). In zebrafish *nbd* mutants and wild-type fish treated with bright light (9000 lux; exposure duration, 48 hours), the photoreceptor cells were degenerated and their behavioral rod and cone sensitivity levels were decreased. In dark-adapted *nbd* and light-treated animals, the rod threshold was −4.61 ± 0.29 and −3.87 ± 0.22 log units, respectively (Fig. 3A). The addition of UV light produced no effect on absolute rod threshold. However, when tested using white light plus luminescence glass-converted green light or nanoparticle-converted white light, increases in rod sensitivity were observed, i.e., the light threshold required to evoke the escape responses decreased to −5.20 ± 0.18 and −5.33 ± 0.19 log units in *nbd* mutants and −4.45 ± 0.20 and −4.60 ± 0.19 log units in light-treated animals (Fig. 3A). In light-adapted *nbd* and light-treated animals, the cone threshold was −3.58 ± 0.21 and −3.71 ± 0.20 log units (Fig. 3B). The addition of UV light produced no statistically significant changes in behavioral cone threshold. However, when UV light was converted to red light or wide-spectrum white light, co-illumination of the fish by white light and converted light resulted in significant increases in cone sensitivity, i.e., the light threshold required to trigger the escape responses decreased to −4.28 ± 0.21 and −4.72 ± 0.18 log units in *nbd* mutants and −4.20 ± 0.19 and −4.61 ± 0.17 log units in light-treated animals (Fig. 3B).

## Discussion

The data demonstrated that the converted light through Tb^3+^ or Eu^3+^ singly-doped glasses or CdS-QDs coated lenses enhances visual performance in zebrafish models. The overall structure and cellular organization of zebrafish retinas are similar to humans, and the physiological functions of zebrafish photoreceptor cells are nearly identical to human photoreceptor cells, i.e., the maximum light absorption of zebrafish and human rod cells is approximately 500 nm wavelength, and in both species the peak absorptive spectra for blue, green, and red cones are 415 nm, 480 nm, and 570 nm, respectively. Note that the zebrafish retinas also contain UV-sensitive cone cells that absorb light at 360 nm^10^,^11^,^12^,^13^. This provides a tool (which also serves as an internal control) for visual measurement in fish illuminated by UV light, either unfiltered or filtered by the singly-doped luminescence glasses and nanoparticle-coated lenses. In developing zebrafish larvae, while the rod and cone system circuitries are still developing, the addition of converted light increased the light absorption of individual photoreceptor cells, thereby enhancing the animals’ visual system functions. The OKR recorded in zebrafish larva when illuminated with solo UV light is likely due to the activation of the UV-sensitive photoreceptor cells and related visual pathways in the retina. It is not triggered by activation of other rod or cone cell types by small components from the UV source. If a small component from UV (e.g., at 500 nm, which may activate the rod photoreceptor cells) have contributed to the OKR, we would expect that the visual thresholds measured under solo UV light are similar at 104 hpf and 128 hpf, because at these developmental stages, the rod photoreceptor cells have been developed. However, the data showed that the threshold levels are different: the threshold is higher in 104 hpf fish than in 128 hpf fish. The data suggest that the OKR detected under UV light is mediated by the activation of the UV cones.

In adult zebrafish, under normal physiological conditions the addition of luminescence glass-converted narrow-band green or red light produced no obvious effect on rod or cone threshold. However, the addition of nanoparticle-converted wide-spectrum white light improved both the rod and cone system functions. In eye-injured animals, the addition of converted light improved the animals’ visual performance. It is likely mediated by the increase of light absorption in the remaining photoreceptor cells.

Based on the results from this research, it is conceivable to propose that Tb^3+^ or Eu^3+^ doped borate glasses or CdS-QDs nanoparticle-coated lenses can be used as an optic devices for visual enhancement in patients who suffer from photoreceptor cell degeneration or other types of eye injuries. For example, by wearing luminescence- or nanoparticle-coated glasses, extra visible light will be produced from UV light emitted from sunlight. This will facilitate retinal functions, thereby restoring some of the lost visual system functions due to photoreceptor cell degeneration or eye injuries. The data described in this paper provide proof-of-concept for the future translational research and the development of optic devices for rod- or cone-photoreceptor cell degeneration patients using luminescence- and/or nanoparticle-related materials.

## Methods

### Glass fabrication, nanoparticle production and coating

Tb^3+^ and Eu^3+^ singly-doped potassium barium borate glasses were made to convert UV light to narrow-band color light. They were prepared using standard melting and quenching techniques. Tb_4_O_7_ (99.99%), Eu_2_O_3_ (99.99%), and analytical reagents K_2_CO_3_, BaCO_3_ and H_3_BO_3_ were used as raw materials. A total of 70g samples were used according to glass molar compositions, and 10K_2_O-20BaO-69B_2_O_3_-1.0Tb_2_O_3_ and 10K_2_O-20BaO-69B_2_O_3_-1.0Eu_2_O_3_ were added to improve material homogeneity. Access boron acid (3mol) was added to compensate the volatile loss during the procedures. Using high pure alumina crucibles, the samples were melted at 1,200 ^o^C for 4 hours in air and then cast onto a cold stainless steel plate to increase the cooling rate and suppress devitrification. After being annealed to release the residual stress inside the glass, the samples were machined and polished.

CdS-polymer nanocomposites were made to convert UV light to white light. Major procedures included the synthesis of water dispersible CdS-QDs, transformation of CdS-QDs into chloroform, and incorporation of CdS-QDs into polystyrene along with POPOP organic dye. Initially, CdS-QDs were synthesized in water using 3-MPA as a surfactant (0.5mmol cadmium chloride was dissolved in 50 ml water followed by the addition of 100 μl 3-MPA). The pH of the solution was adjusted to 10 by NaOH. Then, 0.5mmol thiourea in 10 ml of water was added and refluxed at 90 °C. The solvent was evaporated and the precipitated materials were washed with water-acetone mixture and dispersed in 20 ml water. To transfer the highly water dispersible QDs to organic solvent, 3-MPA was replaced by HVDAC followed by addition of 20 ml CdS-QDs water solution. After 10 min of incubation, the chloroform phase was separated from the water phase using a separating funnel, and CdS-QDs were obtained by evaporating the chloroform. CdS QDs and POPOP were added to the styrene, and then luperox was added for polymerization. Nanocomposites were obtained by sintering at 70 °C for 72 hours.

### Behavioral visual tests

Wild-type, mutant (*nbd*, which displays age-related degeneration of the retina), and light-induced photoreceptor cell degeneration zebrafish models were used in this study. All the experiments involving animals were carried out in accordance with the guidelines approved by the NIH. Animal protocols were approved by the University of Notre Dame IACUC. Visually-mediated animal behaviors were examined to measure the visual sensitivity of developing zebrafish larvae and adult animals. These include the startle responses, optokinetic responses (OKRs), and escape responses. Visual sensitivity was determined by measuring the minimum light that evoked visually-mediated behaviors under white light illumination (Tungsten light; maximum intensity 1200 lux, light intensity was adjusted by adding or removing 0.5 log unit step neutral density filters), either applied alone or in combination of UV light (Deuterium light; 365 or 395 nm wavelength, maximum intensity 50 lux) or luminescence glass- or nanoparticle-converted green, red, or white light. Statistical differences in visual threshold measured before and after the use of converted light were determined by the Student t-test.

To view the startle responses, the larvae were removed from the chorions at 60 hpf, transferred to the Petri dish, and maintained under normal light-dark (14:10) cycles. Each Petri dish hosted only one larva. Prior to the visual test, the larvae were dark adapted for 30 minutes. The stimulus (3-second flashes) was presented to the larva from above. The intensity of the light was initially set at the dimmest level and then increased in 0.5-log unit stops until the first startle responses were observed. Startle responses were defined as abrupt movements in response to light onset or within 2 seconds after light offset. Each larva was tested 5 times. The minimum light that evoked the startle responses in at least 3 out of 5 tests was required to score a light threshold.

The OKR was measured in developing larvae at 104 and 128 hpf. The larvae were immobilized in 1.5% low-melting agarose in the Petri dish. As such, the larvae could not swim around but could freely rotate their eyes. The grating contrast of the strips was set at 100% (black/white). The strips were rotated from either left to right or from right to left at 10 rpm. The strips were illuminated from above and the intensity of the light was initially set at the dimmest level and increased by removing neutral density filters (in 0.5-log unit steps) until the first OKR was observed. Prior to threshold light measurement, the larvae were dark-adapted for 30 minutes. The test lasted 30 seconds, during which time at least 5 positive OKRs (in the direction of strip movement) were required to record a threshold.

The visual sensitivity of adult fish was determined by using an escape response assay. Normally, zebrafish swim slowly along the wall of the container. However, when challenged by a threatening object, i.e., a black segment rotating outside the container, the fish display a robust escape response: as soon as the black segment comes into view, the fish immediately turns and rapidly swims away. By measuring the minimum light required to evoke the escape responses, the visual sensitivity of adult zebrafish can be readily determined. Prior to the test, the fish were dark adapted for 30 min, then the minimum light required to evoke escape responses was recorded. At least 3 positive escape responses out of 5 encounters (between the fish and the rotating black segment) were required to record a threshold light.

## Additional Information

**How to cite this article**: Li, L. *et al.* Luminescence- and nanoparticle-mediated increase of light absorption by photoreceptor cells: Converting UV light to visible light. *Sci. Rep.* **6**, 20821; doi: 10.1038/srep20821 (2016).
